# Metformin attenuates ventilator-induced lung injury

**DOI:** 10.1186/cc11439

**Published:** 2012-07-24

**Authors:** George Tsaknis, Ilias I Siempos, Petros Kopterides, Nikolaos A Maniatis, Christina Magkou, Matina Kardara, Stefania Panoutsou, Anastasia Kotanidou, Charis Roussos, Apostolos Armaganidis

**Affiliations:** 1 GP Livanos and M Simou Laboratories, Evangelismos Hospital, University of Athens-Medical School, Ipsilandou 45-47, Athens, 106 75, Greece; 2 Critical Care Department, Attikon Hospital, University of Athens-Medical School, Rimini 1, Haidari, Athens, 124 62, Greece; 3 Department of Histopathology, Evangelismos Hospital, Ipsilandou 45-47, Athens, 106 75, Greece; 4 First Department of Critical Care and Pulmonary Services, "Evangelismos" Hospital, University of Athens-Medical School, Ipsilandou 45-47, Athens, 106 75, Greece

## Abstract

**Introduction:**

Diabetic patients may develop acute lung injury less often than non-diabetics; a fact that could be partially ascribed to the usage of antidiabetic drugs, including metformin. Metformin exhibits pleiotropic properties which make it potentially beneficial against lung injury. We hypothesized that pretreatment with metformin preserves alveolar capillary permeability and, thus, prevents ventilator-induced lung injury.

**Methods:**

Twenty-four rabbits were randomly assigned to pretreatment with metformin (250 mg/Kg body weight/day *per os*) or no medication for two days. Explanted lungs were perfused at constant flow rate (300 mL/min) and ventilated with injurious (peak airway pressure 23 cmH_2_O, tidal volume ≈17 mL/Kg) or protective (peak airway pressure 11 cmH_2_O, tidal volume ≈7 mL/Kg) settings for 1 hour. Alveolar capillary permeability was assessed by ultrafiltration coefficient, total protein concentration in bronchoalveolar lavage fluid (BALF) and angiotensin-converting enzyme (ACE) activity in BALF.

**Results:**

High-pressure ventilation of the ex-vivo lung preparation resulted in increased microvascular permeability, edema formation and microhemorrhage compared to protective ventilation. Compared to no medication, pretreatment with metformin was associated with a 2.9-fold reduction in ultrafiltration coefficient, a 2.5-fold reduction in pulmonary edema formation, lower protein concentration in BALF, lower ACE activity in BALF, and fewer histological lesions upon challenge of the lung preparation with injurious ventilation. In contrast, no differences regarding pulmonary artery pressure and BALF total cell number were noted. Administration of metformin did not impact on outcomes of lungs subjected to protective ventilation.

**Conclusions:**

Pretreatment with metformin preserves alveolar capillary permeability and, thus, decreases the severity of ventilator-induced lung injury in this model.

## Introduction

Despite its firmly established role as a fundamental life-support modality for critically ill patients, mechanical ventilation (MV) may elicit ventilator-induced lung injury (VILI), which is characterized by alveolar edema and hemorrhage. Recognized mechanisms of VILI include alveolar over-distention by high tidal volumes (volutrauma) and cyclic opening and closing of alveoli (atelectrauma), which operate in concert to trigger inflammatory processes (biotrauma), oxidant/antioxidant imbalance, intra-alveolar coagulation and disturbances in surfactant function [1,2].

Clarification of the above pathophysiologic mechanisms serves a basis for the discovery of effective pharmacological therapies against VILI, which currently is mainly prevented through the limitation of the mechanical insult to the lung parenchyma (that is, through the implementation of protective ventilation) [3]. Such pharmacological therapies could be novel agents, for example, sphingosine 1-phosphate [4], or drugs already in clinical use. Our research group contributed to the idea that established drugs may indeed be beneficial when they are re-used for indications other than their initial indication, a concept which we call the drug recycle concept; indeed, we reported that pretreatment with atorvastatin attenuates VILI [5]. Reduced cost, clinician familiarity and known undesired effects profile are obvious advantages of using drugs with proven efficacy for a different indication.

Such a popular established drug is metformin (N',N'-dimethylbiguanide), which enjoys a long-standing recognition in the setting of type 2 diabetes mellitus. Metformin recently emerged as a potential adjunct in the management of patients with cancer [6-8]; a fact indicating that it may also have effects other than its antihyperglycemic ones. Indeed, there is growing (albeit still limited) evidence that metformin might exhibit pleiotropic properties, including anti-angiogenic [9], anti-inflammatory [10], antioxidant [11] and endothelial barrier-enhancing [12]. Some of the above properties might make metformin a potential candidate for protection against VILI as well.

Interestingly, several clinical studies point out that diabetic patients develop acute lung injury (ALI)/acute respiratory distress syndrome (ARDS) less frequently than non-diabetics [13-15]; a benefit that could be partially ascribed to the usage of antidiabetic therapies, such as metformin [16]. This evidence (derived from observational studies) [13-15] along with the known pleiotropic effects of metformin (derived from experimental studies) [9-12] generated the research idea that it could indeed prevent lung injury. Thus, we endeavored to test the hypothesis that pretreatment with metformin preserves pulmonary vascular permeability, and therefore, confers protection against VILI in non-diabetic animals.

## Materials and methods

### Animal care and pretreatment

A previously described isolated rabbit lung model was implemented [5]. Male New Zealand white rabbits (approximately 3 kg) were used for these experiments, which were conducted in accordance with the 160/1991 Council Directive of European Union. All experimentation was approved by the Institutional Review Board of the Attikon Hospital and by the Veterinary Directorate of the Prefecture of Athens.

Experiment animals were given metformin (250 mg/kg body weight/day *per os *in drinking water) or no medication, 48 hours and 24 hours before surgery.

### Surgical dissection

Detailed information is available in Additional file 1.

### Description of the circuit

#### Perfusate

The perfusate consisted of 350 mL of Krebs-Henseleit solution (to which 5% bovine serum albumin was added) plus 40 mL of autologous blood (serving as a marker for capillary rupture) to achieve a total volume of 390 mL.

#### Circuit

The perfusion circuit consisted sequentially of the left atrium cannula, a venous plastic tubing with its free distal end open to atmosphere, the venous reservoir collecting the perfusate from the venous plastic tubing, a digital rotary pump and an arterial plastic tubing leading consequently to a water bath, a bubble trap and finally to the pulmonary artery cannula and the lungs.

### Initial hemodynamic and ventilation settings

As previously described [5], each heart-lung preparation was connected to a ventilator and the lungs were initially recruited with continuous positive airway pressure (CPAP) of 20 cmH_2_O. Then, each preparation was ventilated with pressure-control ventilation (PCV) at peak inspiratory pressure of 11 cmH_2_O, positive end-expiratory pressure (PEEP) of 3 cmH_2_O, respiratory rate of 15 breaths/minute, inspiration:expiration ratio of 0.5 and fraction of inspired oxygen of 0.21, while the blood flow increased gradually to 300 mL/minute. Heart-lung preparations were maintained in these conditions (that is, constant perfusion with 300 mL/minute and mechanical ventilation with PCV 11:3) for the next 16 minutes and were subsequently switched to CPAP 5 cmH_2_O to measure the ultrafiltration coefficient (K*f,c*) [17,18].

### Group allocation and ventilation/perfusion protocol

After measurement of the baseline K*f,c*, each lung preparation was randomly allocated to be ventilated with PCV at peak inspiratory pressures of 23 cmH_2_O (high pressure, HiP) or 11 cmH_2_O (low pressure, LoP) for 60 minutes. Four experimental groups of lung preparations were set: high pressure metformin pretreatment (HiP-Met); high pressure no metformin (HiP-C); low pressure metformin pretreatment (LoP-Met) and low pressure no metformin (LoP-C). Experiments were not performed in a blinded manner.

### Post-ventilation protocol measurements

After the completion of the ventilation protocol and the measurement of the final K*f,c*, the blood flow and ventilation were stopped. The following variables were considered as outcomes for our study:

ΔKf,c (that is, final K*f,c *- baseline K*f,c*) was the change in K*f,c *before and after the institution of the 60-minute ventilation protocol. ΔK*f,c *indicated pulmonary capillary permeability alterations. Weight gain was the weight of the heart-lung preparation at 20, 40 and 60 minutes minus its weight after 1 minute of the ventilation protocol. Given that the weight of the heart could not change during the ventilation, this variable reflected the change in lung weight and, eventually, it served as a surrogate for formation of pulmonary edema. Changes in pulmonary artery pressure (mean, inspiratory and expiratory) were defined as the pulmonary arterial pressure at 20, 40 and 60 minutes minus the pulmonary arterial pressure after 1 minute of the ventilation protocol. Changes in tidal volume were defined as the tidal volume at 20, 40 and 60 minutes minus the tidal volume after 1 minute of the ventilation protocol. Total protein concentration in bronchoalveolar lavage fluid (BALF) was measured, as previously described [5].

Angiotensin-converting enzyme (ACE) activity in BALF was measured following incubation of BALF with ACE substrate hippuryl-histidine-leucine and using a previously described fluorometric assay [19]. Increased levels of ACE activity in BALF indicated diffusion of this enzyme into the alveolar spaces and, thus, served as a marker of pulmonary microvascular barrier disruption [19].

The total cell number in BALF was calculated using a hemocytometer.

### Histology

The left lung was fixed for histology and a composite histological score was determined as previously described [5,20,21].

### Statistical analysis

GraphPad Prism 5 (La Jolla, CA, USA) was used for statistical analyses. Data were summarized as means ± standard deviation. One way analysis of variance was used to determine the statistical significance of between-group differences. Statistically significant results (*P *< 0.05) were further examined by post hoc analysis using the Student Newman-Keuls test. For outcomes such as BALF protein, BALF ACE activity, BALF total cell number and histology, where data from four animals per group were available, data were summarized as medians (range) and compared using the non-parametric Kruskal-Wallis test.

## Results

Of the thirty-two animals sacrificed for this experiment, eight animals were omitted before the measurement of the baseline K*f,c *according to prespecified exclusion criteria. Thus, lung preparations from twenty-four rabbits (specifically, seven animals per HiP group and five animals per LoP group) were used.

No difference was found between the compared groups in serum glucose level at baseline (Table 1). HiP groups (that is, HiP-Met versus HiP-C) did not differ in terms of baseline characteristics (Table 1). At the beginning of the mechanical ventilation, HiP groups exhibited higher tidal volume than LoP groups (HiP 16.5 ± 1.9 mL/kg vs. LoP 6.8 ± 1.5 mL/kg). With respect to the values of perfusate pH, arterial oxygen tension (PaO_2_) and arterial carbon dioxide tension (PaCO_2_), HiP-Met and HiP-C groups did not differ at any time of ventilation (data provided in Additional file 1).

**Table 1:** Characteristics of the compared groups at baseline.

Variables	LoP-C	LoP-Met	HiP-C	HiP-Met
Animal weight, kg	3.2 ± 0.2	3.4 ± 0.2	3.3 ± 0.4	3.0 ± 0.3
Serum glucose, mg/dL	179 ± 44	182 ± 91	152 ± 93	144 ± 59
Initial lung weight, g	24.1 ± 2.4	24.4 ± 2.3	23.8 ± 2.5	20.6 ± 2.7
Ischemic time, minutes	34.6 ± 5.5	39.0 ± 7.1	39.0 ± 6.8	34.4 ± 4.9
**Before the beginning of 60-minute ventilation**				
PPAmean, mmHg	20.4 ± 3.5	24.8 ± 7.5*	16.8 ± 3.1*	16.7 ± 4.0*
PPAinspir, mmHg	24.9 ± 3.8	30.8 ± 8.4	22.5 ± 4.4	22.0 ± 4.4
PPAexpir, mmHg	18.4 ± 3.7	21.4 ± 6.7*	13.9 ± 2.7*	12.9 ± 2.2*
Pcap, mmHg	7.5 ± 1.8	7.0 ± 0.6	6.3 ± 0.4	6.8 ± 0.6
K*f,c*, g/min/mmHg/100g	0.185±0.09	0.147±0.05	0.209±0.07	0.244±0.11
**At the beginning of 60-minute ventilation:**				
pH	7.33 ± 0.17	7.27 ± 0.09	7.32 ± 0.11	7.33 ± 0.07
PaO_2_, mmHg	161 ± 9**	154 ± 9	147 ± 2**	147 ± 8
PaCO_2_, mmHg	44.7 ± 19.9	47.2 ± 6.6	44.8 ± 5.4	44.7 ± 7.3
T, °C	35.9 ± 0.5	35.2 ± 0.8	36.2 ± 0.8	35.6 ± 0.5

LoP-C: low pressure-no metformin; LoP-Met: low pressure-metformin; HiP-C: high pressure-no metformin; HiP-Met: high pressure-metformin; PPAmean: mean pulmonary arterial pressure; PPAinspir: inspiratory pulmonary arterial pressure; PPAexpir: expiratory pulmonary arterial pressure; Pcap: pulmonary capillary hydrostatic pressure; K*f,c*: ultrafiltration coefficient; PaO_2_: arterial oxygen tension; PaCO_2_: arterial carbon dioxide tension; T: temperature. Results are presented as means ± standard deviation; n = 7 animals per HiP group and 5 animals per LoP group. *P < 0.01 for comparison between the LoP-Met and both HiP groups; **P < 0.05 for comparison between the LoP-C and HiP-C group.

### Pulmonary capillary permeability (K*f,c*)

**Figure 1:**
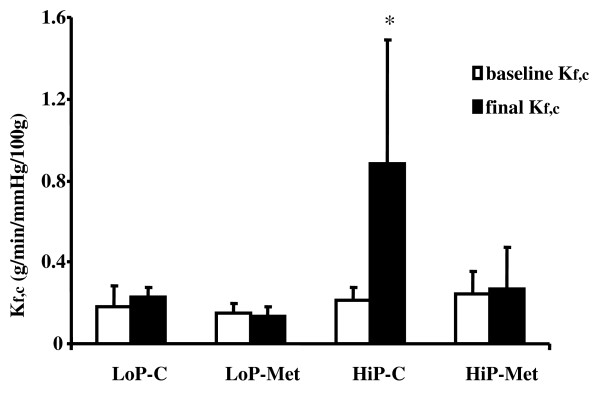
**Ultrafiltration coefficient measured at baseline (baseline K*f,c*) and after the ventilation protocol (final K*f,c*)**. Results are presented as means ± standard deviation; n = 7 animals per HiP group and 5 animals per LoP group. *P < 0.05 for HiP-C group vs. all other groups. LoP-C: low pressure-no metformin; LoP-Met: low pressure-metformin; HiP-C: high pressure-no metformin; HiP-Met: high pressure-metformin.

At baseline, compared groups did not differ with regard to K*f,c *(expressed in g/min/mmHg/100 g) (Table 1). At the end of ventilation, final K*f,c *was greater in the HiP-C group than in both the LoP-C and LoP-Met groups, but there was no difference between the HiP-Met and either of the LoP groups (Figure 1).

Among lungs ventilated at the higher peak inspiratory pressure/higher tidal volume, pretreatment with metformin compared to no pretreatment was associated with lower final K*f,c *(HiP-Met 0.267 ± 0.205 vs. HiP-C 0.881 ± 0.605) (Figure 1) and lower ΔΚ*f,c *(HiP-Met 0.023 ± 0.266 vs. HiP-C 0.672 ± 0.613).

### Pulmonary edema formation (weight gain)

**Figure 2:**
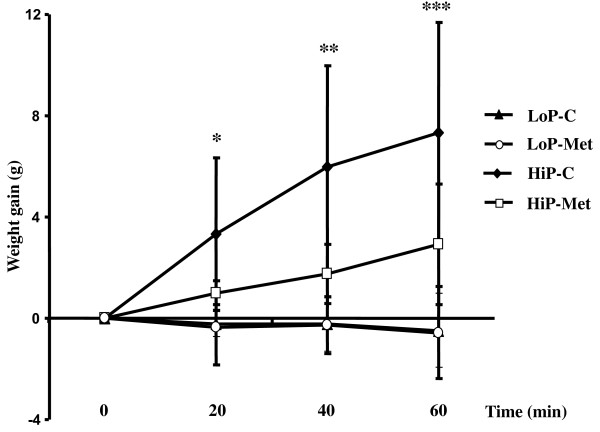
**Weight gain at different time points during the ventilation protocol**. Results are presented as means ± standard deviation; n = 7, 6 and 5 animals for HiP-C, HiP-Met and LoP groups, respectively. *P < 0.05 for HiP-C group vs. all other groups; ** *P *< 0.01 for HiP-C group vs. all other groups; *** *P *< 0.05 for HiP-C group vs. HiP-Met and *P *< 0.001 for HiP-C group vs. LoP groups. LoP-C: low pressure-no metformin; LoP-Met: low pressure-metformin; HiP-C: high pressure-no metformin; HiP-Met: high pressure-metformin.

The HiP-C group developed more edema than both LoP groups at 20, 40 and 60 minutes of ventilation (Figure 2), but the HiP-Met group did not differ from either LoP group (Figure 2).

Among lungs ventilated at the higher peak inspiratory pressure/higher tidal volume, those with, as opposed to those without metformin pretreatment, sustained less weight gain at 20 minutes (HiP-Met 1.01 ± 0.47 g vs. HiP-C 3.32 ± 3.02 g), 40 minutes (HiP-Met 1.76 ± 1.18 g vs. HiP-C 5.96 ± 4.01 g) and 60 minutes (HiP-Met 2.91 ± 2.38 g vs. HiP-C 7.35 ± 4.32 g) of mechanical ventilation (Figure 2). This was also the case after adjustment of weight gain for initial lung weight.

### Changes in pulmonary arterial pressure

There was no difference between the compared groups in increase in mean pulmonary arterial pressure at any time of ventilation. This was also the case for the expiratory and the inspiratory pulmonary pressure.

### Changes in tidal volume

**Figure 3:**
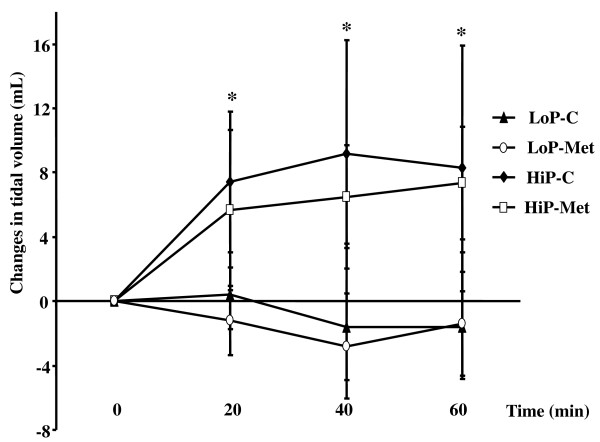
**Changes in tidal volume at different time points during the ventilation protocol**. Results are presented as means ± standard deviation. n = 7, 6 and 5 animals for HiP-C, HiP-Met and LoP groups, respectively. *P < 0.05 for HiP groups vs. LoP groups. LoP-C: low pressure-no metformin; LoP-Met: low pressure-metformin; HiP-C: high pressure-no metformin; HiP-Met: high pressure-metformin.

As shown (Figure 3), there was a greater increase in tidal volume in the HiP groups than in the LoP groups at all ventilation times. In contrast, there was no difference between the HiP-Met and HiP-C groups.

### Gross examination of the lungs

After the end of injurious ventilation, we noticed more edema and more hemorrhagic spots on the surface of the lungs exposed in injurious (HiP) compared to protective (LoP) ventilation. Among the HiP groups, on gross observation there was less edema and fewer hemorrhagic spots on the lungs of animals with metformin pretreatment (HiP-Met group) as opposed to those without (HiP-C group).

### Protein concentration in BALF

**Figure 4:**
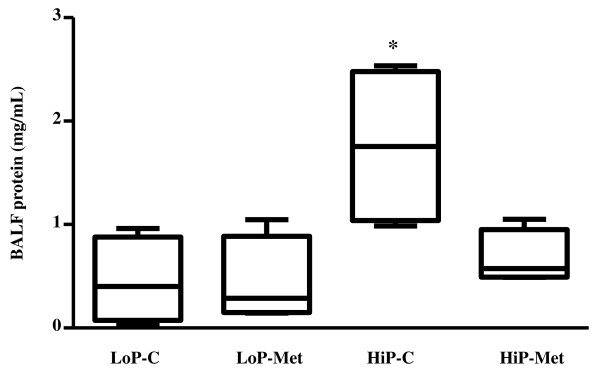
**Total protein concentration in bronchoalveolar lavage fluid (BALF)**. Results are presented as medians (range); n = 4 animals per group. *P < 0.05 for HiP-C group vs. other groups. LoP-C: low pressure-no metformin; LoP-Met: low pressure-metformin; HiP-C: high pressure-no metformin; HiP-Met: high pressure-metformin.

Among the HiP groups, those with metformin pretreatment had lower total protein concentration in BALF compared to those without (HiP-Met 0.57, range 0.49 to 1.05 mg/mL vs. HiP-C 1.75, range 0.99 to 2.53 mg/mL) after the end of 60 minutes of ventilation (Figure 4).

### ACE activity in BALF

**Figure 5:**
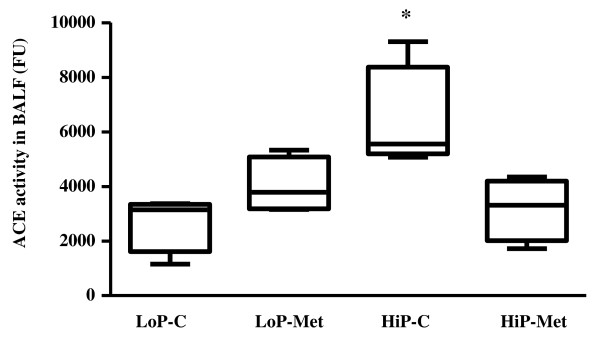
**Angiotensin-converting enzyme (ACE) activity in bronchoalveolar lavage fluid (BALF)**. Results are presented as medians (range); n = 4 animals per group. *P < 0.05 for HiP-C group vs. HiP-Met and LoP-C groups. LoP-C: low pressure-no metformin; LoP-Met: low pressure-metformin; HiP-C: high pressure-no metformin; HiP-Met: high pressure-metformin; FU: fluorescence units.

After the end of injurious ventilation, animals pretreated with metformin (HiP-Met) presented lower levels of ACE activity in BALF compared to untreated animals (HiP-C) (Figure 5).

### Total cell number in BALF

There was no difference between the compared groups in the total cell number in BALF.

### Histology

**Figure 6:**
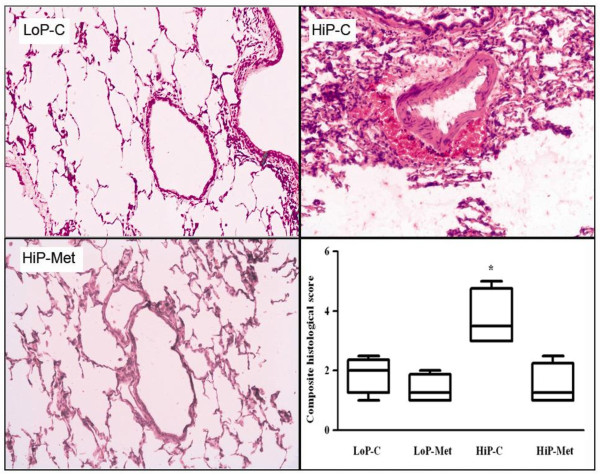
**Histological lesions due to mechanical ventilation**. Representative histological sections show less perivascular hemorrhage in metformin-pretreated (HiP-Met) compared to untreated (HiP-C) animals after exposure to one hour of injurious ventilation. Perivascular hemorrhage was not observed in the lungs of animals exposed to protective ventilation (LoP-C). Results are presented as medians (range); n = 4 animals per group. *P < 0.05 for HiP-C group vs. all other groups. LoP-C: low pressure-no metformin; LoP-Met: low pressure-metformin; HiP-C: high pressure-no metformin; HiP-Met: high pressure-metformin.

In lungs ventilated at the higher peak inspiratory pressure/higher tidal volume, metformin pretreatment, as opposed to no pretreatment, was associated with fewer histological lesions in terms of perivascular hemorrhage (HiP-Met 0, range 0 to 0) vs. HiP-C 2, range 0 to 2) and composite histological score (HiP-Met 1.25, range 1.0 to 1.5) vs. HiP-C 3.5, range 3.0 to 5.0) (Figure 6). In contrast, no difference was demonstrated between the HiP-Met and HiP-C groups in capillary congestion and infiltration of alveolar spaces by neutrophils. We did not observe intra-alveolar hemorrhage, interstitial infiltration, hyaline membrane formation or thickening of the basal membrane in any of the groups.

## Discussion

The findings of the present study suggest that pretreatment with metformin prevented increases in pulmonary microvascular permeability in response to injurious mechanical ventilation, thus attenuating VILI in terms of edema formation and histology.

One may wonder if this beneficial effect of metformin against VILI could be attributed to the modification of the serum glucose level of treated animals rather than the metformin itself. The evidence (albeit controversial) that hyperglycemia promotes ALI might justify this notion [13,22]. However, serum glucose levels did not differ in the groups compared in our study. This was congruent with the results of other experiments that also showed that administration of metformin in non-diabetic animals did not alter their glycemic status [10]. Similarly, administration of metformin does not influence serum glucose in humans who are not suffering from diabetes mellitus [23]. Thus, metformin appears to prevent VILI by a mechanism other than its antihyperglycemic action.

By measuring the pulmonary microvascular filtration coefficient K*f,c*, an accurate marker of pulmonary microvascular permeability [24,25], in addition to BALF protein and ACE activity levels, we observed that metformin prevented the rise in endothelial permeability upon challenge of the isolated lung preparation with excessive airway pressure, indicating a direct barrier-stabilizing effect of the drug. In line with our results, other investigators have advocated that metformin restores pulmonary endothelial function after injurious stimuli other than over-distention, such as hypoxia, through activation of adenosine monophosphate-activated protein kinase [12]. Interestingly, beneficial effects of metformin on the endothelial function of organs other than lung were repeatedly reported [26,27], indicating that metformin might act similarly on the pulmonary vascular endothelium as well, hence justifying further research in the field.

Agard and colleagues reported that metformin exhibits vasodilatory effects and therefore, it protects against experimental pulmonary hypertension [12]. This finding [12], in conjunction with evidence regarding the impact of pulmonary hypertension (combined with high airway pressure) on the development of lung injury [28], could lead to the claim that mitigation of VILI in our study could take place through improvements in hemodynamics. However, we found no difference between the metformin (HiP-Met) and the no metformin (HiP-C) groups in pulmonary arterial pressure at any time of injurious ventilation, and given the constant-flow conditions and pulmonary venous pressure of our system, no differences in pulmonary vascular resistance can be expected between the groups. Thus, the putative vasodilatory effects of metformin (as indicated by the determination of pulmonary arterial pressure in the HiP groups) appear not to contribute to the attenuation of lung injury under the present experimental conditions.

Likewise, other pleiotropic effects of metformin, such as anti-inflammatory effects [10], may also not participate in its benefit against VILI in the present study. Our experimental protocol had a negligible amount of leucocytes in the perfusate and a short duration of injurious ventilation, so there was not enough time for inflammatory pathways to be activated. Indeed, we found no difference between the compared groups in terms of the BALF total cell number and infiltration of alveolar spaces by neutrophils. Thus, in our model of VILI (in which mechanical phenomena rather than inflammation are the major determinants of injury), it seems unlikely that a drug could provide benefit by acting as an anti-inflammatory agent.

A comment on our results of the changes in tidal volume during the 60-minute ventilation period may be worthwhile (Figure 3). Indeed, we observed an increase in the tidal volume in lungs ventilated at the higher peak inspiratory pressure/higher tidal volume (HiP groups) during the early phases of injurious ventilation, which (given the applied PCV mode of ventilation) could be translated into an increase in compliance (Figure 3). This increase in compliance after the initiation of high pressure ventilation is a consistent finding in our *ex-vivo *experiments [5,20,21]. On the other hand, as depicted in Figure 3, during the last 20 minutes of the injurious ventilation, lungs in the HiP-C, but not the HiP-Met group, sustained a decrease in tidal volume (and thus, in compliance). This could be explained by the formation of pulmonary lung edema in the HiP-C group as VILI proceeded.

Careful combing of the literature may reveal several (indirect) indications that usage of metformin might prevent ALI. Specifically, during the last few years, there is accumulating clinical evidence that a medical history of diabetes mellitus predicts reduced susceptibility to ALI/ARDS in patients at risk. Indeed, at least three large cohorts of patients with risk factors for ALI/ARDS (namely sepsis/septic shock, trauma and massive transfusion) inferred that there is a protective association between diabetes and ALI/ARDS, even after adjustment for potential confounders [13-15]. In a relevant review, it was assumed that diabetes may merely be a confounder and instead other factors related to the management of diabetic patients, such as antihyperglycemic therapies, may account for the observed protection of such patients [16]; the lack of firm evidence that acute hyperglycemia (a cardinal feature of diabetes) alone modulates ALI makes this assumption reasonable [13,29]. Metformin is a very commonly prescribed anti-diabetic medication; thus, it may mediate the relative protection of diabetic patients against the development of ALI.

Our isolated lung model, albeit well-established for the research of VILI, entails unavoidable limitations. First, it is an *ex-vivo *model, which means that lungs do not interact with other organs or the lymphatic or the nervous system. However, this model provides us with the ability to accurately measure the K*f,c *and, thereby, to reliably assess the improvements in alveolar capillary permeability achieved by the administration of metformin [24,25]. In addition, it allows us to continuously monitor the formation of edema, and pulmonary arterial pressure over time.

Second, although our *ex-vivo *rabbit lung model provided us with the ability to make an intriguing observation (namely, metformin preserves alveolar capillary permeability despite injurious ventilation), it could not allow us to gain a mechanistic insight into how metformin affords this benefit. For example, we were unable to examine whether metformin protects through inhibition of the mitochondrial function and oxidative burst of leukocytes [30]. There is evidence that metformin induces a hypometabolic state [31], which in turn might be beneficial against injury [32]. Additional *in-vivo *animal studies are justified, focusing on the mechanism through which metformin prevents VILI.

Third, our choice to administer a dose of metformin as high as 250 mg/kg/day and not to check for actual drug intake might be questioned. Rare reports that metformin is associated with lactic acidosis, which is surprisingly of good prognosis [33], might justify this criticism. However, the risk for lactic acidosis due to metformin seems to be overestimated according to a recent relevant meta-analysis [34]. Given that we did not measure pH and lactate concentration in the blood of rabbits before sacrificing them, we could not preclude subclinical lactic acidosis. However, we did not observe any apparent adverse event after administration of the drug. Similarly, several other investigators who gave the same dosage of metformin (250 mg/kg) did not notice any toxicity [10,35].

Fourth, data on several outcomes (namely BALF protein, BALF ACE activity, BALF total cell number and histology) were derived from only four animals per group. In an attempt to address this concern, we treated these data conservatively; specifically, we summarized them as the median and the range and compared these using non-parametric tests. Finally, we noticed that our HiP-C group sustained less injury than in our previous study [5]. However, given that all groups (HiP-Met, HiP-C, LoP-Met and LoP-C) in the present study were compared during the same time period and under the same experimental conditions (that is, without the use of historical controls), this could not affect the robustness of our results.

## Conclusions

The findings of the present study demonstrate that pretreatment with the widely used antidiabetic agent metformin protects against VILI under the present experimental conditions. These results expand our knowledge regarding the non-antidiabetic effects of metformin and may be of clinical value. Should these results be replicated in an *in-vivo *animal model of VILI, they might provide a rationale for carrying out observational studies that will examine the relationship between administration of metformin and development of ALI/ARDS. The fact that metformin is inexpensive and already widely used may make execution of such trials feasible.

## Key messages

• Oral administration of metformin does not cause hypoglycemia or other apparent adverse events in non-diabetic rabbits.

• Administration of metformin decreases the severity of VILI.

## Abbreviations

ACE: angiotensin-converting enzyme; ALI: acute lung injury; ARDS: acute respiratory distress syndrome; BALF: bronchoalveolar lavage fluid; CPAP: continuous positive airway pressure; ΔK*f,c*: change in ultrafiltration coefficient*; *HiP: high pressure; HiP-C: high pressure-no metformin; HiP-Met: high pressure-metformin pretreatment; K*f,c*: ultrafiltration coefficient; LoP: low pressure; LoP-C: low pressure-no metformin; LoP-Met: low pressure-metformin pretreatment; MV: mechanical ventilation; PaCO_2_: arterial carbon dioxide tension; PaO_2_: arterial oxygen tension; PCV: pressure-control ventilation; PEEP: positive end-expiratory pressure; VILI: ventilator-induced lung injury.

## Competing interests

The authors declare that they have no competing interests.

## Authors' contributions

GT carried out the experiments. IIS conceived the study, participated in its design, carried out the experiments, interpreted the data and drafted the manuscript. PK and NAM aided study design, data interpretation and manuscript correction. CM performed the histological analysis. MK and SP performed the biochemical assays. AK and CR aided study design. AA conceived the study, participated in its design and corrected the manuscript. All authors read and approved the final manuscript.

## Supplementary Material

Additional file 1 **Detailed methods, and table giving detailed information regarding animal care and pretreatment, surgical dissection, description of the circuit, measurement of baseline ultrafiltration coefficient, exclusion criteria, group allocation and ventilation/perfusion protocol, post-ventilation protocol measurements and histology**. Characteristics (pH, arterial oxygen tension and arterial carbon dioxide tension) of the compared groups at different time points during the 60-minute ventilation period are depicted. Click here for file

## References

[B1] Dreyfuss D Saumon G Ventilator-induced lung injury: lessons from experimental studies Am J Respir Crit Care Med 1998 157 294 323 9445314 10.1164/ajrccm.157.1.9604014

[B2] Tremblay LN Slutsky AS Ventilator-induced injury: barotrauma to biotrauma Proc Assoc Am Physicians 1998 110 482 488 9824530

[B3] The Acute Respiratory Distress Syndrome Network Ventilation with lower tidal volumes as compared with traditional tidal volumes for acute lung injury and the acute respiratory distress syndrome N Engl J Med 2000 342 1301 1308 10793162 10.1056/NEJM200005043421801

[B4] Jacobson JR Pharmacologic therapies on the horizon for acute lung injury/acute respiratory distress syndrome J Investig Med 2009 57 870 873 10.2310/JIM.0b013e3181c04681 19820408

[B5] Siempos II Maniatis NA Kopterides P Magkou C Glynos C Roussos C Armaganidis A Pretreatment with atorvastatin attenuates lung injury caused by high-stretch mechanical ventilation in an isolated rabbit lung model Crit Care Med 2010 38 1321 1328 20308883 10.1097/CCM.0b013e3181d9dad6

[B6] Currie CJ Poole CD Jenkins-Jones S Gale EA Johnson JA Morgan CL Mortality after incident cancer in people with and without type 2 diabetes: impact of metformin on survival Diabetes Care 2012 35 299 304 10.2337/dc11-1313 22266734 PMC3263862

[B7] Tan BX Yao WX Ge J Peng XC Du XB Zhang R Yao B Xie K Li LH Dong H Gao F Zhao F Hou JM Su JM Liu JY Prognostic influence of metformin as first-line chemotherapy for advanced nonsmall cell lung cancer in patients with type 2 diabetes Cancer 2011 117 5103 5111 10.1002/cncr.26151 21523768

[B8] Ben Sahra I Le Marchand-Brustel Y Tanti JF Bost F Metformin in cancer therapy: a new perspective for an old antidiabetic drug? Mol Cancer Ther 2010 9 1092 1099 10.1158/1535-7163.MCT-09-1186 20442309

[B9] Tan BK Adya R Chen J Farhatullah S Heutling D Mitchell D Lehnert H Randeva HS Metformin decreases angiogenesis via NF-kappaB and Erk1/2/Erk5 pathways by increasing the antiangiogenic thrombospondin-1 Cardiovasc Res 2009 83 566 574 10.1093/cvr/cvp131 19414528

[B10] Zmijewski JW Lorne E Zhao X Tsuruta Y Sha Y Liu G Siegal GP Abraham E Mitochondrial respiratory complex I regulates neutrophil activation and severity of lung injury Am J Respir Crit Care Med 2008 178 168 179 10.1164/rccm.200710-1602OC 18436790 PMC2453511

[B11] Morales AI Detaille D Prieto M Puente A Briones E Arévalo M Leverve X López-Novoa JM El-Mir MY Metformin prevents experimental gentamicin-induced nephropathy by a mitochondria-dependent pathway Kidney Int 2010 77 861 869 10.1038/ki.2010.11 20164825

[B12] Agard C Rolli-Derkinderen M Dumas-de-La-Roque E Rio M Sagan C Savineau JP Loirand G Pacaud P Protective role of the antidiabetic drug metformin against chronic experimental pulmonary hypertension Br J Pharmacol 2009 158 1285 1294 10.1111/j.1476-5381.2009.00445.x 19814724 PMC2782337

[B13] Moss M Guidot DM Steinberg KP Duhon GF Treece P Wolken R Hudson LD Parsons PE Diabetic patients have a decreased incidence of acute respiratory distress syndrome Crit Care Med 2000 28 2187 2192 10921539 10.1097/00003246-200007000-00001

[B14] Gong MN Thompson BT Williams P Pothier L Boyce PD Christiani DC Clinical predictors of and mortality in acute respiratory distress syndrome: potential role of red cell transfusion Crit Care Med 2005 33 1191 1198 10.1097/01.CCM.0000165566.82925.14 15942330

[B15] Iscimen R Cartin-Ceba R Yilmaz M Khan H Hubmayr RD Afessa B Gajic O Risk factors for the development of acute lung injury in patients with septic shock: an observational cohort study Crit Care Med 2008 36 1518 1522 10.1097/CCM.0b013e31816fc2c0 18434908

[B16] Honiden S Gong MN Diabetes, insulin, and development of acute lung injury Crit Care Med 2009 37 2455 2464 10.1097/CCM.0b013e3181a0fea5 19531947 PMC3103784

[B17] Townsley MI Korthuis RJ Rippe B Parker JC Taylor AE Validation of double vascular occlusion method for Pc,i in lung and skeletal muscle J Appl Physiol 1986 61 127 132 3733597 10.1152/jappl.1986.61.1.127

[B18] Hotchkiss JR Jr Blanch L Murias G Adams AB Olson DA Wangensteen OD Leo PH Marini JJ Effects of decreased respiratory frequency on ventilator-induced lung injury Am J Respir Crit Care Med 2000 161 463 468 10673186 10.1164/ajrccm.161.2.9811008

[B19] Maniatis NA Letsiou E Orfanos SE Kardara M Dimopoulou I Nakos G Lekka ME Roussos C Armaganidis A Kotanidou A Inhaled activated protein C protects mice from ventilator-induced lung injury Crit Care 2010 14 R70 10.1186/cc8976 20403177 PMC2887192

[B20] Kapetanakis T Siempos II Metaxas EI Kopterides P Agrogiannis G Patsouris E Lazaris AC Stravodimos KG Roussos C Armaganidis A Metabolic acidosis may be as protective as hypercapnic acidosis in an ex-vivo model of severe ventilator-induced lung injury: a pilot study BMC Anesthesiol 2011 11 8 10.1186/1471-2253-11-8 21486492 PMC3087686

[B21] Kopterides P Kapetanakis T Siempos II Magkou C Pelekanou A Tsaganos T Giamarellos-Bourboulis E Roussos C Armaganidis A Short-term administration of high oxygen concentration is not injurious in an ex-vivo rabbit model of ventilator-induced lung injury Anesth Analg 2009 108 556 564 10.1213/ane.0b013e31818f10f7 19151287

[B22] Yilmaz M Keegan MT Iscimen R Afessa B Buck CF Hubmayr RD Gajic O Toward the prevention of acute lung injury: protocol-guided limitation of large tidal volume ventilation and inappropriate transfusion Crit Care Med 2007 35 1660 1666 10.1097/01.CCM.0000269037.66955.F0 17507824

[B23] Borst SE Snellen HG Metformin, but not exercise training, increases insulin responsiveness in skeletal muscle of Sprague-Dawley rats Life Sci 2001 69 1497 1507 10.1016/S0024-3205(01)01225-5 11554611

[B24] Parker JC Townsley MI Evaluation of lung injury in rats and mice Am J Physiol Lung Cell Mol Physiol 2004 286 L231 246 10.1152/ajplung.00049.2003 14711798

[B25] Bhattacharya J Interpreting the lung microvascular filtration coefficient Am J Physiol Lung Cell Mol Physiol 2007 293 L9 L10 10.1152/ajplung.00148.2007 17468133

[B26] Wang J Alexanian A Ying R Kizhakekuttu TJ Dharmashankar K Vasquez-Vivar J Gutterman DD Widlansky ME Acute exposure to low glucose rapidly induces endothelial dysfunction and mitochondrial oxidative stress: role for AMP kinase Arterioscler Thromb Vasc Biol 2012 32 712 720 10.1161/ATVBAHA.111.227389 22207730 PMC3319449

[B27] Sena CM Matafome P Louro T Nunes E Fernandes R Seiça RM Metformin restores endothelial function in aorta of diabetic rats Br J Pharmacol 2011 163 424 437 10.1111/j.1476-5381.2011.01230.x 21250975 PMC3087142

[B28] Hotchkiss JR Jr Blanch L Naveira A Adams AB Carter C Olson DA Leo PH Marini JJ Relative roles of vascular and airspace pressures in ventilator-induced lung injury Crit Care Med 2001 29 1593 1598 10.1097/00003246-200108000-00016 11505134

[B29] Wasmuth HE Kunz D Graf J Stanzel S Purucker EA Koch A Gartung C Heintz B Gressner AM Matern S Lammert F Hyperglycemia at admission to the intensive care unit is associated with elevated serum concentrations of interleukin-6 and reduced ex vivo secretion of tumor necrosis factor-alpha Crit Care Med 2004 32 1109 1114 10.1097/01.CCM.0000124873.05080.78 15190958

[B30] Protti A Fortunato F Monti M Vecchio S Gatti S Comi GP De Giuseppe R Gattinoni L Metformin overdose, but not lactic acidosis per se, inhibits oxygen consumption in pigs Crit Care 2012 16 R75 10.1186/cc11332 22568883 PMC3580617

[B31] Protti A Russo R Tagliabue P Vecchio S Singer M Rudiger A Foti G Rossi A Mistraletti G Gattinoni L Oxygen consumption is depressed in patients with lactic acidosis due to biguanide intoxication Crit Care 2010 14 R22 10.1186/cc8885 20170489 PMC2875537

[B32] Protti A Singer M Bench-to-bedside review: potential strategies to protect or reverse mitochondrial dysfunction in sepsis-induced organ failure Crit Care 2006 10 228 10.1186/cc5014 16953900 PMC1751057

[B33] Vecchio S Protti A Metformin-induced lactic acidosis: no one left behind Crit Care 2011 15 107 10.1186/cc9404 21349142 PMC3222034

[B34] Salpeter SR Greyber E Pasternak GA Salpeter EE Risk of fatal and nonfatal lactic acidosis with metformin use in type 2 diabetes mellitus Cochrane Database Syst Rev 2010 4 CD002967 20091535 10.1002/14651858.CD002967.pub3

[B35] Zou MH Kirkpatrick SS Davis BJ Nelson JS Wiles WG 4th Schlattner U Neumann D Brownlee M Freeman MB Goldman MH Activation of the AMP-activated protein kinase by the anti-diabetic drug metformin in vivo. Role of mitochondrial reactive nitrogen species J Biol Chem 2004 279 43940 43951 10.1074/jbc.M404421200 15265871

