# Supplementary material for: Metformin attenuates ventilator-induced lung injury
Source: Crit Care. 2012 Jul 24;16(4):R134. doi: 10.1186/cc11439 (PMC3580719; doi:10.1186/cc11439)
Supplement: Additional file 1 — Detailed methods, and table giving detailed information regarding animal care and pretreatment, surgical dissection, description of the circuit, measurement of baseline ultrafiltration coefficient, exclusion criteria, group allocation and ventilation/perfusion protocol, post-ventilation protocol measurements and histology. Characteristics (pH, arterial oxygen tension and arterial carbon dioxide tension) of the compared groups at different time points during the 60-minute ventilation period are depicted. [file cc11439-S1.DOC]

**Additional file 1**

Metformin attenuates ventilator-induced lung injury

George Tsaknis, MD, Ilias I. Siempos, MD, Petros Kopterides, MD, Nikolaos A. Maniatis, MD, Christina Magkou, MD, Matina Kardara, PhD, Stefania Panoutsou, PhD, Anastasia Kotanidou, MD, Charis Roussos, MD, Apostolos Armaganidis, MD

# METHODS

# Animal care and pretreatment

The rabbits were housed in single metal cages, on a 12:12-hour light/dark cycle, at room temperature of approximately 20^o^C, and had free access to food.

**Surgical dissection**

Anesthesia was induced by intramuscular injection of ketamine (1 mL/Kg) and xylazine (0.5 mL/Kg), and maintained with continuous intravenous infusion of thiopental (10 mg/Kg/h). After placement of a tracheotomy tube, animals were sternotomized and exsanguinated through laceration of the inferior vena cava. The heart-lung preparation was excised *en block* and weighed. Perfusion cannulas were placed in the pulmonary artery and in the left atrium via incision in the free wall of the right and of the left ventricle, respectively. Then, the heart-lung preparation was suspended by the endotracheal tube from a counterbalanced force transducer connected with a balance, which continuously monitored preparation weight.

**Description of the circuit**

*Perfusate:* The perfusate hematocrit was 3%, its white blood cell count was negligible and its temperature was maintained at 36^o^C through a water bath. A gas mixture of carbon dioxide (35-45%) and room air (65-55%) was continuously insufflated into the perfusate to control perfusate pH and arterial carbon dioxide tension (PaCO_2_).

*Data recording:* Pressure and weight signals were amplified, stored on a personal computer via an analog-digital converter and analyzed by software.

**Measurement of baseline K*f,c***

Measurement of baseline K*f,c*, was made as previously described [1,2]. Briefly, after preparation weight had stabilized (achievement of isogravimetric conditions during CPAP of 5 cmH_2_O and constant blood flow of 300 mL/min), pulmonary capillary hydrostatic pressure was measured in duplicate by simultaneous double occlusion. Then, the weight was allowed to stabilize for 5 min, and the free end of the left atrial cannula rapidly raised to obtain a left atrial pressure of 12 mmHg. After continuous recording of preparation weight for 7 min, pulmonary capillary hydrostatic pressure was again measured in duplicate, and left atrial pressure was returned to 6 mmHg. K*f,c* (in g/min/mmHg/100g of lung tissue) was calculated by dividing the rate of weight gain between the 5^th^ and 7^th^ min following the elevation of the left atrial pressure by the increase in the pulmonary capillary hydrostatic pressure and was finally normalized to the initial lung weight.

**Exclusion criteria**

In accordance with our previous experiments [1, 2], the following exclusion criteria were posed: presence of gas bubbles or emboli into the tubes of the perfusion circuit, air leak or hemorrhagic lesions on the lungs’ surface, perfusate leakage, and mean pulmonary artery pressure higher than 25 mmHg before the measurement of the baseline K*f,c*.

**Group allocation and ventilation/perfusion protocol**

All heart-lung preparations were perfused constantly (300 mL/min) and ventilated with positive end-expiratory pressure: 3 cmH_2_O, respiratory rate: 15/min, inspiration:expiration ratio: 0.5 and fraction of inspired oxygen: 0.21. At prespecified time points (namely at the beginning, 20^th^ min, 40^th^ min and 60^th^ min) of the ventilation, a small amount of perfusate was aspirated and used for measurement of perfusate pH, PaCO_2_ and arterial oxygen tension (PaO_2_). During the 60 min of the ventilation protocol, the free end of the left atrial cannula was fixed to a height corresponding to a left atrial pressure of 6 mmHg. Thus, all heart-lung preparations (regardless of group) had a left atrial pressure of 6 mmHg during the ventilation period.

**Postventilation protocol measurements**

*Total protein concentration and total cell number in bronchoalveolar lavage fluid (BALF):* After the completion of the ventilation protocol, the right lung of each preparation was lavaged by infusion of 15 mL of normal saline twice. Four mL of the recoverd BALF were merged with eight mL of a methanol based, buffered preservative solution (CytoLyt, Cytyc Corporation, MA, USA) and were later used for calculation of total cell nuber with hematocytometer. The remaining BALF was centrifuged (800 *g* for 10 min at 4^o^C) and its supernatant was used for the measurement of BALF protein concentration, as previously described [1].

**Histology**

After placing a ligature around the right hilum, only the left lung was fixed for histology by instillation of a 10% formaldehyde solution (30 mL). The lung was cut into slices 5 mm thick in a coronal fashion from apex to base. Only two specimens from the left lung (specifically one specimen from the dorsal field of upper lobe and another specimen from the dorsal field of lower lobe) of each animal were obtained, embedded in paraffin, stained with hematoxylin and eosin, and examined by an experienced pathologist. Lung injury was scored according to the presence of 1) infiltration of neutrophils in the alveolar spaces, 2) interstitial infiltration of neutrophils, 3) perivascular and 4) intra-alveolar hemorrhage, and 5) capillary congestion, as previously described [1]. Each item was scored according to a five-point scale (namely 0: minimal or no damage, 1: mild damage, 2: moderate damage, 3: severe damage, and 4: maximal damage). A composite histological score including all the above items was also determined [1].

**REFERENCES**

1. Siempos II, Maniatis NA, Kopterides P, Magkou C, Glynos C, Roussos C, Armaganidis A: **Pretreatment with atorvastatin attenuates lung injury caused by high-stretch mechanical ventilation in an isolated rabbit lung model.** *Crit Care Med* 2010, **38**:1321-1328.
2. Kapetanakis T, Siempos II, Metaxas EI, Kopterides P, Agrogiannis G, Patsouris E, Lazaris AC, Stravodimos KG, Roussos C, Armaganidis A: **Metabolic acidosis may be as protective as hypercapnic acidosis in an ex-vivo model of severe ventilator-induced lung injury: a pilot study.** *BMC Anesthesiol* 2011; **11**:8.

**RESULTS**

| **Table S1. Characteristics of the compared groups at different time points during the 60 min ventilation period.** | | | | |
| --- | --- | --- | --- | --- |
| **Variables** | **LoP-C** | **LoP-Met** | **HiP-C** | **HiP-Met** |
|  |  |  |  |  |
| ***At 20 min of ventilation:*** | | |  |  |
| pH | 7.29±0.09 | 7.33±0.11 | 7.32±0.12 | 7.32±0.06 |
| PaO_2_, mmHg | 156±9* | 154±6 | 145±6* | 147±6 |
| PaCO_2_, mmHg | 44.9±5.9 | 46.4±8.8 | 51.0±14.4 | 44.3±5.2 |
|  |  |  |  |  |
| ***At 40 min of ventilation:*** | | |  |  |
| pH | 7.31±0.10 | 7.32±0.09 | 7.28±0.09 | 7.34±0.12 |
| PaO_2_, mmHg | 156±6 | 152±6 | 144±7 | 146±8 |
| PaCO_2_, mmHg | 42.4±5.5 | 40.6±4.9 | 53.9±10.7 | 46.4±13.1 |
|  |  |  |  |  |
| ***At the end of 60 min ventilation:*** | | |  |  |
| pH | 7.29±0.09 | 7.32±0.04 | 7.37±0.14 | 7.26±0.12 |
| PaO_2_, mmHg | 151±6 | 150±12 | 145±16 | 148±10 |
| PaCO_2_, mmHg | 44.4±7.1 | 42.4±6.6 | 44.7±5.3 | 50.2±20.0 |

*Definitions of abbreviations:* LoP-C: low pressure-no metformin, LoP-Met: low pressure-metformin, HiP-C: high pressure-no metformin, HiP-Met: high pressure-metformin, PaO_2_: arterial oxygen tension, PaCO_2_: arterial carbon dioxide tension. Results are presented as means ± standard deviation. *n*= 7 animals per HiP group and *n*= 5 animals per LoP group.

* p< 0.01 between LoP-C group and HiP-C group.
